# Supplementary material for: Design considerations for a hierarchical semantic compositional framework for medical natural language understanding
Source: PLoS One. 2023 Mar 16;18(3):e0282882. doi: 10.1371/journal.pone.0282882 (PMC10019629; doi:10.1371/journal.pone.0282882)
Supplement: Attachment — Submitted filename: Response to Reviewers.docx [file pone.0282882.s001.docx]

**Response to Reviewers**

The authors would like to sincerely thank the reviewers for their efforts in providing critical and helpful comments for this paper submission. We have substantially added to the Discussion section to provide a detailed perspective regarding the major issues brought up during the review process. Below we address each point of concern from each of the reviewers.

# Reviewer #1

The paper described a design of a semantic model and an associated parser used to transform a free-text sentence into a logical representation of the meaning of clinical text. The main contribution of this paper is to enhance machine understanding clinical text semantically.

**--------------------------------------**

**Question 1**: While the four-point framework aims to provide a structure for designing NLU system, some points had relatively similar concepts in natural language processing, e.g. both “Predefined Semantic Representations” and “Semantic Activation Networks” are both similar to knowledge models in medical knowledgebases and ontologies. Overall, the paper lacks comparison to other NLP concepts, tasks and systems.

## Response 1:

Thank you for this comment. We have added a paragraph in the Discussion Section to explain how current NLP and knowledge engineering tasks fit into the architecture. We have also added a reference to our prior work entitled “Hierarchical semantic structures for medical NLP,” that summarizes the role of traditional NLP methods and knowledge sources within the context of the goals of the HSCM architecture. The table shows the relationship between the following: semantic layers, state space of nodes, the mapping tasks between semantic layers, the sub-problems associated with each mapping class, common knowledge sources employed within each layer, algorithms and tools associated with various subtasks, and global optimization methods. Additionally, within the paragraph, we contrast the current processing scheme with a traditional NLP pipeline. Throughout the revised manuscript, we have also been more mindful to clarify the distinction and similarities of such components within the described framework. Besides, there are several major problems:

**--------------------------------------**

**Question 2**. The structure of the paper is confusing, “we first introduce the overall NLU problem highlighting the need for a predefined compositional structure” – this should be defined in the Introduction.

## Response 2:

We have moved this text excerpt into the Introduction as suggested.

**--------------------------------------**

**Question 3**: The author should also highlight major deliverable of the paper “Hierarchical Semantic Compositional Model (HSCM)” earlier in the manuscript as well as in the abstract.

## Response 3:

We have modified the Abstract and Introduction to bring out this point.

**--------------------------------------**

**Question 4**: Definition of “surface words” is not clear.

## Response 4:

We have defined its meaning in the text (viz., the verbatim string).

**--------------------------------------**

**Question 5**: How is Semantic Layer 4-6 different from each other?

## Response 5:

We have added additional text within the manuscript to distinguish these levels. In summary, they represent fundamentally informational templates of differing degrees of semantic abstraction details. Level 4, the propositional level, concerns basic semantic relations. These propositions can include locative predicates (e.g., “extends laterally from location A to location b), temporal predicates, and descriptive predicates of clinical findings (e.g., has size 5cm). Level 5 contains specific ontologic templates (frames) that are intended to capture the full complex state description of an object or event that serves as the frame head. For example, at this level, we have developed a comprehensive ontology for a mass, that includes all the properties (about 200) used in radiology to characterize a mass. Event templates (e.g., a radiology procedure) can include attributes such as date, patient, departments, time/date, etc.), or in general argument such as AGENT, THEME, RECIPENT, etc. At Level 6, this level includes discourse models between the speaker and listener (e.g., event scripts). An example of a discourse model topic would be the expected communicative goals of a radiologist describing a patient’s smoking habits in the context of determining whether a patient is eligible for lung cancer screening. In particular, this discourse model has been used to disambiguate instances of ellipsis commonly observed in this domain (incomplete specification of units of pack-years, which can be implicated from the discourse model).

**--------------------------------------**

**Question 6**: “This structure enables a more efficient process of encoding sentence meaning, by facilitating a generative model.” – how do you measure efficiency? Do you compare your parser with other semantic parsers?

## Response 6:

As this is a concept paper, we did not perform any experiments related to implementation efficiency.

We focused on overarching principles and architectural organization required to achieve a deep level of understanding, leaving specific methodological implementations open. However, this statement was made on two theoretical grounds: composition (representational efficiency) and hierarchal predictive coding (processing efficiency). Imposing a compositional structure (i.e., factorization) is known to contribute significantly to reducing the dimensionality (i.e., computational complexity) of the parsing problem [see references by Geman 2002 and Yuille 2013]. It provides a framework for ‘part sharing’ which allows development to proceed in a piece-wise systematic way. (Of course, the up-front cost of such an approach is in curating this HSCM knowledge source for the target clinical application). As described in Yuille, this part sharing can lead to an enormous reduction in complexity.

Predictive coding offers processing efficiency since only plausible hypotheses specified within the HSCM grammar need be tested. A combination of top-down (hypothesis formulation) and bottom up (hypothesis testing) processing conducted within a hierarchical predictive paradigm greatly reduces the search state space for a viable global sentence parse. Note that a purely bottom up (inverse problem) approach to semantic parsing is regarded as an ill-posed problem [Berton 1987]. The HSCM model provides the additional constraints in order to rule out a large number of interpretation possibilities.

In practice, we have implemented this processing scheme using finite state grammars with stored memory patterns, which makes the general approach extremely efficient. Grammars that implement dynamic programming algorithms also are known to be efficient. We will report specific performance measures in a follow up paper describing the system’s ability to perform full semantics parses of sentences describing tumoral masses from radiology reports.

S. Geman, D. Potter, and Z. Chi. Composition systems. Quarterly of Applied Mathematics, 60(4):707–736, 2002.

Yuille, A. L. and Mottaghi, R. (2013) ‘Complexity of representation and inference in compositional models with part sharing’, *1st International Conference on Learning Representations, ICLR 2013 - Conference Track Proceedings*, pp. 1–13.

Barton EG, Berwick RC, and Ristad ES. Computational Complexity and Natural Language, Bradford Books, Cambridge, MA, MIT Press, 1987.

**--------------------------------------**

**Question 7**: “Our design involves explicitly defining this structure in a way that parallels the manner in which humans compose meaning.” – How is your methods different from other NLP tasks that extract information from unstructured clinical text and create a structured output?

## Response 7:

Thank you for this question. We have included text in the discussion section to highlight the main differences compared to current information extraction systems and what the new capabilities it provides. The main difference is the trend towards a cognitive architecture. Thus, the proposed architecture introduces the concepts of semantic priming, semantic frames, situational ontologies, discourse micro-theories, hierarchical predictive coding, and distributed agent-based processing which are currently not discussed in mainstream medical NLP research. These ideas provide an NLU system with a semantic substrate for integrating heterogeneous knowledge sources allowing improved context for making local NLP decisions, for integrating multiple modes of inference, and a well-defined path toward continued system development via the notion of composition and part-sharing.

# Reviewer #2:

This manuscript proposes a new framework for NLU (natural language understanding) based mostly on semantic analysis (semantic memory, composition, activation and hierarchical predictive coding). This interesting framework is based on a few human cognition characteristics and contrasted with current deep learning-based approaches. The manuscript is very well written, but the framework description remains quite vague in several aspects and leaves most details and possible implementations to be defined. This vagueness might keep it more flexible and technology-agnostic but also makes understanding several aspects of the proposed framework quite difficult.

**Response**: As you have pointed out, the paper is vague with respect to implementation details. Our goal was to emphasize architectural and processing paradigm issues that we feel should be considered as a long-term strategic direction for building medical NLU systems. The technologies used to implement the pieces of the design are open, so we felt for this introductory paper, that we leave the descriptions required for implementation at the specification level. We are currently working on two implementation papers describing utilizing the framework for applications related to understanding radiology mass descriptions and patient smoking habits.

**Major Compulsory Revisions**:

The following revisions are recommended:

**--------------------------------------**

**Question 8**: The framework proposes an approach that is presented as applicable to all clinical narratives and their possible content, but also mention that circumscribing the scope of this possible semantic content is part of the modeling process. This is a critical issue and balance to find between a very specific semantic field specific to one specialty, note type, institution and even individual author, and broad biomedical and general knowledge. More discussions around this issue and strategies to address it should be added.

## Response 8:

Indeed, the aspects of wide-coverage and comprehensive understanding are at odds. We have added text in the Discussion section to address this point. We summarize the discussion below..

a) Semantic Specificity - The problems that required natural language “understanding” (i.e., intended meaning) are tied to patient outcomes. One important distinction within an the medical informatics field, is that the NLU system’s worth hinges on providing the necessary information to accomplish an “actionable” task. Medical NLU system’s thus are not intended to be general broad coverage agents, but rather targeted agents that are tasked to understand text at sufficient levels of detail and content to correctly guide a clinical or research action. It is important to realize that these tasks can be high-stake and/or mission critical responsibilities, compared to an NLP system that may be searching for information stored in the web or in journal articles. Thus, ignoring tail distribution cases is often unacceptable. For example, we have worked on applications for identifying patients who should be screened for lung cancer. Failure to identify such patients should not be hinged on idiosyncratic language used in a medical report. Having a logical sematic substrate to integrate pragmatic and global situational models will improve an NLU ability to handle complex language phenomenon (e.g., ellipses, coreference resolution, and linguistic paraphrasing).
b) Coverage – This aspect of NLU should proceed with the efforts of a community of contributors and performed steadily and carefully over time. The HSCM semantic framework is designed to reflect how humans perceive the world and thus provides a grounded semantic substrate from which we can build a community-agreed upon representation. As with BERT models, the HSCM model is intended to be a sharable resource that can be deployed for an ever-expanding number of medical NLU applications. The logical ontological and semantic compositional aspects of the model should be fairly universal and stable across languages. Because the grammars are localized to a HSCM node, independent developers should be able to contribute to the core model. An important community effort would then be to provide the required tools to keep with the principles of the design. In a follow-up implementation paper, we will report on a set of initial tools we have developed to implement the described system.
c) Adaptability between specialty, note type, institutions, author, and languages – Building language models can be said to be a continuous endeavor given the generally wide variety of expressions, and the many linguistic issues that often are seen in real-world clinical text. The HSCM model is intended to include finely granular concepts and information templates. Therefore, although the number of nodes are numerous, each node results in a relatively low dimensional grammar. For a given node (e.g., smoking predicate frame), we can gather sentences containing the verb ‘smoking’ across many domains and institutions. Diversity sampling methods for learning the semantic arguments and grammars can provide consistent coverage for a wide genre of text (i.e., clinical text from various procedures, institutions and authors) [Flickinger 2011].
d) Scalability is another benefit of a semantic compositional approach to encoding sentence meaning [Bender 2015]. It is realized via the reuse of developed components, built across applications. For example, with minimal manual knowledge engineering, an NLU application that can capture the state of a tumoral mass can share its common properties (e.g., size, shape, radiographic intensity features) with an HSCM node that describes an aneurysm using the object-oriented aspect of abstraction.

Flickinger, D. Accuracy vs. robustness in grammar engineering. In E. M. Bender and J. E. Arnold (Eds.), Language from a Cognitive Perspective: Grammar, Usage, and Processing, pp. 31–50. Stanford: CSLI Publications, 2011.

Bender EM, Flickinger D, Oepen S, and Packard W. Layers of interpretation: on grammar and compositionality. In: Proceedings of the 11th International Conference on Computational Semantics; 2015 April 15-17; London, United Kingdom. 2015. p. 239-249

**--------------------------------------**

**Question 9**: As mentioned above, the complexity of certain aspects of the proposed framework and the vague description make understanding sometimes difficult. A few punctual examples are provided, but a more complete and realistic example (maybe broader than the radiology report examples provided) used throughout the different steps of the proposed NLU process would significantly help understand the concepts proposed.

## Response 9:

We have added a few more clarifying examples from smoking to clarify complex language modeling phenomenon, including semantic implicature.

**--------------------------------------**

**Question 10**: Among desirable characteristics of NLU/NLP frameworks, generalizability is among the top and is a very current problem, but mostly ignored in your manuscript; it is not even mentioned in its list of qualities. On page 11, you state that you favor topic-centric and corpus-based approaches (with specific note types); this strategy might allow for better performance in this specific field, but also tends to generalize to other domains with difficulty.

## Response 10:

We have added a paragraph in the discussion section that addresses issues related to the balance between fine-grained comprehension and general applicability. Given that the HSCM is a frame-based hierarchical ontologic representation of the semantic constituents covered by the system, many of the benefits achieved by such designs foundational technologies can be realized. For example, the frame-based design allows HSCM node construction to benefit from object-oriented principles such as inheritance, abstraction, modularity, and encapsulation. Ontologic design principles are used to generalize activation networks during parsing execution. Composition allows for sharing of constituent parts. See also response 8 for discussion of generality.

**--------------------------------------**

**Question 11**: Word sense disambiguation is an important step in the semantic analysis of clinical text, and how it could be implemented remains unclear. Would it be part of semantic layer 1 (e.g., for abbreviations expansion)? Or another one for eponyms for example? Similarly, analysis of the local context of the information/concepts identified (e.g., negation) is critical but not clearly part of your semantic constituents; layer 4? Or 5?

## Response 11:

Although we did not mention these tasks specifically, they are all tasks that are inclusive within the framework. We have included discussions of each of these tasks within the text in order to clarify how the framework handles them. The issues raised (word sense disambiguation, negation, abbreviation expansion, and characterization of eponyms) are all handled by the node specific construction methods (e.g., grammars) for each semantic constituents within the HSCM. (We implement locally context sensitive grammars for each node).

Context for identifying an instance within the HSCM model comes from both general language models and from observed sentence features. General ontologic knowledge is encapsulated within a node and can define syntactic structural constraints and semantic selectional restrictions. At the sentence level, context integration is a processor issue, and is handled by the hierarchical predictive coding algorithm. The agent based architecture and the level managers use locally context-sensitive grammars as well as can integrate global context such as procedure type or situational context models (e.g., reporting of smoking habits for lung cancer screening). Note that the context for interpretation is also facilitated by the architecture’s semantic priming step used within the predictive coding paradigm.

**--------------------------------------**

**Question 12**: The discussion contracting the proposed framework with deep learning-based approaches using word embeddings etc. is very important nowadays and should be expanded with more structure and concrete examples. Demonstrating the proposed superiority of your approach would rely on it.

## Response 12:

We note that deep learning methods and the current approach are not mutually exclusive. We need to be careful how we use deep learning methods and understand their strengths and weaknesses. For example, we mention in the text that word embeddings are in fact a feature of a sentence word token. Other features include morphological features, part-of-speech, possible dictionary semantic senses, etc. Therefore, any higher-level concept proposed by the predictive coding algorithm has access to any and all such features.

We have included in the Discussion that the system is open to any algorithm that can perform a specified symbol-to-symbol mapping within the HSCM, whether rule based, finite state, statistical, or deep learning. In fact, the agent-based architecture allows multiple algorithms to be attached to test a hypothesized HSCM concept during parsing. Also, graph embeddings could be used to generalize FSM grammars, and/or propose new nodes and connects within the HSCM itself.

**--------------------------------------**

Minor Essential Revisions:

**Question 13**: Stating that automated deep understanding of clinical notes remains elusive and generally far from human abilities is probably correct, but requires evidence (e.g., cited bibliographic references) and would benefit from a more detailed description of specific limitations your proposed framework would address.

## Response 13:

Thank you for this question. NLU system must be able to infer speaker intent in contexts where the language has been abbreviated (e.g., ellipsis). We have included a reference citing the fundamental lack of mechanisms that prevent current deep learning methods from theoretically ever achieving this level of comprehension. In particular the reference by Bender, which was awarded the Best Theme paper at the 2020 Association for Computational Conference, explains the limits of deep learning NLP architectures. The theme of the conference centered around the question: “Are current methods really enough to achieve the field’s ultimate goals?”

Bender EM and Koller A. Climbing toward NLU: on meaning, form, and understanding in the age of data,” Proceedings of the 58^th^ Annual Meeting of the Association for Computational Linguistics, (Best Theme Paper), Pages 5185-5198, July 2020.

**--------------------------------------**

**Question 14**: On page 5 last line, “lexical level” would match the other levels mentioned better than “at the level of words”.

## Response 14:

Thank you. We have made this suggested correction.

**--------------------------------------**

**Question 15**: On page 32, stating that the clinical language is more restrictive than “open-ended” text might be true for radiology reports but has been demonstrated as less restrictive in other domains and note types. Some clarification is needed.

## Response 15:

We have clarified this statement. Restrictions of text within clinical medical informatics applications is notably driven by the goal of an “actionable interpretation” (see Dunietz 2020 reference also). Thus, even though other note types have more diverse topics and language styles, the target text required to correctly make an actionable interpretation is still quite narrow. Within this task definition, the context is much constrained compared to considering all possible sentence forms from all possible types of clinical text for all possible interpretations. We have included a discussion of the range of writing styles in clinical report to try to clarify the domain a bit more clearly. In general, clinical text can range from highly grammatical (e.g., declarative styles in radiology;), to abbreviated styles in ophthalmology notes, to episodic descriptions from discharge summaries; and from lay and slang language from the chief complains described in the patient’s own words. We also comment on writing styles of novice physicians (e.g., residents), vs experienced physicians (compiled vs first principles) [Langlotz 2015].

Dunietz J. “The field of natural language processing is chasing the wrong goal,” MIT Technology Review, July, 31, 2020.

Langlotz CP. The radiology report: a guide to thoughtful communication for radiologists and other medical professionals. 2015, ISBN: 978-1515174080

# Reviewer #3:

It is refreshing to see described, a proposed system design drawing from well-researched theories in the cognitive sciences, and practical considerations in the medical imaging reporting domain, that attempts to performs knowledge synthesis and inference similar to specialized human experts. This is in sharp contrast to many leading conference and medical NLP workshop papers in recent times employing the latest state of the art deep learning methods, but all of which read very much the same.

This work could definitely be poised to become a unique and important contribution in the area of medical NLU. However, there are some key aspects of completeness that must be addressed first.

**--------------------------------------**

**Question 16**: The main thrust of the article seems to be around the premise that "compositionality" plays a key role in the human-like understanding by machines, of complex sentence structure, stating perhaps rightly that we would not lose any information given in the sentence by factoring it into components that are themselves meaningful at various levels of semantic abstraction. These components seemingly could even be at the level of ontological primitives and propositions besides characters, tokens or phrases. However, little is presented in terms of evidence or experiments to support this claim or hypothesis.

## Response 16:

Thank you for this question. Our intent is to follow-up this concept design paper with a two proof-of-concept papers that describe performance measures associated with implementing the ideas of the this work within two application domains (tumor mass descriptions and patient smoking habits). In the revision, we have included an anecdotal example of how compositionality can be used to facilitate the development of an application for aneurysms utilizing semantic constituents developed for tumoral mass descriptions. We have also expanded the Discussion section of the paper to bring forward some of the well reported advantages of compositionality reported in the literature.

The basis for this statement on compositionality was made from two long standing principles in linguistics: 1) Bottom-up: Principle of Composition – that the meaning of the whole sentence is a function of the meaning of its parts [Montague, Cresswell 1973, Dummett 1973, Fodor 1992]; and 2) Top-Down: Context Principle – that words have meaning only as constituents of the sentence [Dummett 1973]. Fillmore described language understanding from the perspective of semantic frames and the idea that contextual regularities could be encapsulated in a grammar [Fillmore 1985]. Our design incorporates these ideas by proposing the use of semantic frames for all levels of tokens, by attaching grammars for each constituent within the HSCM, and by hierarchical incremental parsing to improve context within each processing stage. Figure 4 shows the incremental synthesis of semantic constituents that finally are aggregated into a unifying sentence level semantic frame (see Figure 4).

From a computational AI point of view, we have added the references from Geman 2002 and Yuille 2013 as discussed in the response to Question 6 (from Reviewer 1) above. This response was in reference to computational efficiencies gained.

Finally, there has been much discussion within the AI community related to what types of knowledge are required for systems to truly generalize beyond their training data. Central to these discussions is the need for compositionality [See Tenenbaum 2011].

1. Fillmore, C. J. (1985). Frames and the semantics of understanding. Quaderni di Semantica, 6(2), 222-254, 1985
2. Pelletier FJ. Compositionality and Concepts—A Perspective from Formal Semantics and Philosophy of Language Compositionality and Concepts in Linguistics and Psychology, Volume 3, 2017
3. Dummett M.  *Frege: Philosophy of Language*, Cambridge, MA: Harvard University Press, 1973.
4. Fodor J. and LePore E. (1992). Holism: A Shopper’s Guide, Oxford: Blackwell, 1992
5. Tenenbaum JB, Kemp C, Griffiths TL, and Goodman ND. “How to grow a mind: statistics, structure, and abstraction,” Science 331:1279-1285, 2011.

**--------------------------------------**

**Question 17**: The introduction and background sections give the reader a solid overview of the different elements that will go into the design of the framework, such as semantic composition, semantic activation networks and hierarchical predictive coding. However, the paper structure, while it is meant to be about the design considerations for such a framework, lacks the structure of a clear hypothesis and supporting (even preliminary) experiments, analysis, or initial prototype that serves to "inform" such a design.

## Response 17:

In this paper, our goal was to make an argument for a cognitive architecture. We feel it is first necessary to explain to the medical informatics community the conceptual framework and the underlying foundational principles. Although we have constructed a working prototype of the design, presenting the technical details of the implementation would distract from the conceptual arguments made in this design consideration paper.

We are planning to report in the near future on various experiments conducted on two applications built from an initial prototype implementation of the architecture (radiology mass descriptions and patient smoking history).

In the Conclusion section of the paper, we restate these main issues for the reader.

**--------------------------------------**

**Question 18**. It is good to see models such as BERT, BioBERT cited in references, however the necessary comparisons as to how the proposed framework is at least "different" if not better, than these, by way of just some simple examples or analysis is lacking. E.g. if the authors are familiar with the recent work "BERT rediscovers the classical NLP pipeline." by Tenney et al. that source could have been cited and appropriate comparisons presented. E.g. this aforementioned paper talks about how internal BERT architecture layers are found to perform increasingly complex tasks, viz. part-of-speech tagging, parsing, entity recognition, semantic roles, and finally coreference, in that order, towards NLU. This is reminiscent of the various level managers in Figure 4 of this draft. Thus a simple comparison by means of a chart or a table between the framework in Figure 4, and BERT, could have been provided, highlighting side-by-side, how existing language models of the day such as BERT, or its fine-tuned variants like BioBERT process and understand language at each level, compared to the proposed HSCM framework. This might give the reader an immediate intuition of how and why HSCM can be expected to perform better and perhaps produce more human-like inferences. At the least it would highlight the benefits and drawbacks of each paradigm.

# Response 18:

Thank you for your question and suggestions. We have included a table and corresponding discussion comparing and contrasting a BERT model with the HSCM knowledge source. Four dimensions of comparison are presented: 1) description of the layers; 2) Intended Use of models; 3) Effort – what is the human cost function; 4) Capabilities and Potential for long term growth and utility. This comparison is presented in the Discussion section as motivation for building the HSCM model as a stable long-term resource that can support diverse inferencing methods.

**Description of layers** - The paper by Tenney describes that when probing the a BERT transformer model, one can conclude that qualitatively the internal layers seem to be encoding raw language properties of input text such as part-of-speech tags, syntactic constituents, syntactic dependencies, semantic roles, co-references, and prototype roles. Transformer models such as BERT have become the state of the art with respect to its application to specific medical NLP problems. The layers of the BERT model, as you point out, are seemingly similar to the HSCM layers. Although, BERT does indeed show these abilities to identify various language specific properties, relations and constituents, these mappings are made in a fuzzy statistical manner based on word associations (distributed semantics). The control parameters available to users would be the corpus, the dimensions of the transform layers, and training related parameters. What this shows is that a hierarchical encoding of language into various constituents can provide a principled means of analyzing language. In the case of the HSCM, the layering is based on semantic composition that reflects how humans perceive language. For example, there is general agreement of how one might create predicate-argument structures, or how a radiologist might define a semantic frame describing the properties of a mass. In BERT models, there is no grounding of any of the constituents to real world ontologic definitions, although in some cases they can approximate this mapping. Given the incredible applicability of BERT for many NLP problems however, it is clear that a sharable high quality language encoding knowledge source can be a core resource for many language processing tasks. We feel in the long term, a core knowledge source such as the described HSCM that provides semantically rich, transparent interpretations using ontologic components will better satisfy the requirements for medical text NLU applications. Since the HSCM is grounded to ontologic semantic constituents, it can integrate various heterogeneous ontologies to extend its logical inferencing abilities.

**Intended Use** – The HSCM knowledge is intended for building precise, ontologically grounded interpretations of narrow domain text. For example, in building a phenotype description of imaging findings described in radiology reports or in building a database of current patient smoking behavior. As the model matures over time, it can take on a greater range of application since the semantic constituents are sharable across applications.. Its semantic substrate provides the methodological hooks for integrating precise language micro-theories that can be used to improve global context for text understanding. BERT models on the other hand, are intended to be general knowledge sources for natural language processing tasks that can be addressed with language sequence models and with the precision offered by a distributed semantic representation.

**Development Effort** – Curating knowledge sources into precompiled representations that are design specifically for actionable medical informatics applications requires significant efforts. Compared to BERT based programs, the current architecture requires very detailed ontologic modelling and will require continued efforts to model the grammars of each of the semantic constituents. These efforts will be an ongoing process of development and refinement. We believe that within an applied field such as medical informatics, curating such precise semantic models and grammars should be emphasized given the often critical nature of the NLU task.

**Capabilities and Potential for Long-term growth** – The logical HSCM model is relatively stable, assuming the developers adhered to the underlying principles of composition and authoring ontologies. This ontologic view for representing meaning should generally agree across the medical informatics community and with the logical structure even agreeable across many world languages. The agent-based architecture can provide controls for how an application navigates through the HSCM as well as provides flexibility with respect to importing pragmatic context, integration of features, inferencing mechanisms, and fallback planning. This provides a long-term framework for integrating discourse and pragmatic knowledge into the text understanding process. Another important feature of the HSCM knowledge source is the transparency it provides for meaning synthesis, which facilitates debugging, incremental structured development, and user acceptance.

We have added additional text within the Discussion section of the paper, including Table 2, to summarize these points, as suggested by the reviewer.

**--------------------------------------**

**Question 19**: Also, while the implementation details of each piece of the framework has been left to the user, it is not clear what sort of time complexity considerations are at play, for encoding and decoding/inference functions of the framework, e.g. steps 1,2,3 and 4 of Figure 5. This may lead the reader to assume deep learning-based systems are at a greater advantage, at a fraction of the representation, training and inference cost. Some estimate of what time and space complexity each layer/piece of the framework involves by way of some simple example, might give the reader a better idea.

## Response 19:

Thank you for this question. We have included in the paper new references for readers to explore with respect to the time complexity issues associated with predictive coding and semantic composition. The time complexity of course depends on the specific implementations for each mapping step. The main reduction however, is realized by the predictive coding step guided by the HSCM semantic substrate. A full theoretical discussion of how predictive coding can readily solve seemingly ill-posed problems (mapping all possible input signals to all possible interpretations) using the free-energy theory, is provided in the paper by Friston “Active Inference: a process theory”. One should also note that the efficiencies gained by a neural network using backpropagation learning is closely approximating an implementation of predictive coding [see Whittington 2017, and Song 2020].

Efficiency is also brought about due to the extensive use of fine-grain composition of constituents that allows the overall NLU problem to be factored into a number of lower dimensional mappings. We reference two publications [Yuille 2013 and Poggio 2015] that discuss theoretical complexity assessments associated with compositional models.

Whittington JCR and Bogacz R. “An approximation of the error backpropagation algorithm in a predictive coding network with local Hebbian synaptic plasticity,” Neural Comput 29(5):1229-1262, 2017.

Song Y, Lukasiewicz, Xu Z and Bogacz R. “Can the brain do backpropagation? --- Exact implementation of backpropagation in predictive coding networks,” Proceedings of the NeurIPS, 2020

Yuille AL, Mottaghi R. “Complexity of Representation and Inference in compositional models and part sharing, International Conference on Learning Representations (ICML). 2013

Poggio T, Anselmi F, Rosasco L. “I-theory on depth vs width: hierarchical functional composition,” CBMM Memo 041, Center for Brains Minds and Machines. Massachusetts Institute of Technology, December 2015.

**--------------------------------------**

**Question 20:** The claim of greater interpretability possible with the HSCM framework and how it arrives at final decisions in terms of "true intended meaning" of a parse has not been entirely elaborated either.

## Response 20:

We have included a paragraph in the Discussion section describing interpretability issues and have included a few relevant references.

The question of “true intended meaning” within NLP systems has remained nebulous even within the discipline of natural language understanding. The recent paper by Dunietz 2020, proposes a framework for evaluating comprehension. The main issue relates to how well an NLU system can answer the necessary queries related to the driving application. However, the important question to be asked for true understanding involves not only did the system get the right answer, but did it access the right content for how it derived the answer, rather than using some spurious correlations seen in the data. This issue is especially relevant with respect to an NLU’s acceptance by the medical informatics community.

The HSCM model is intended to address application queries using inferences made in the logical ontologic world, rather than the linguistic text world. The HSCM model provides transparency with respect to the path within the HSCM model used to derive a parse of a sentence. This transparency is thus the source of explanation offered by the system. We have included in the paper illustrations of this transparency and explanatory abilities of the design using an example from an NLU smoking application which required integrating both sentence level features and domain knowledge related to a medical screening protocol to correctly interpret the intentions of the authoring physician.

1. Dunietz J, Burnham G, Bharadwaj A, Rambow O, Chu-Carroll J, and Ferrucci D. “To test machine comprehension, start by defining comprehension,” Proceedings of the 58^th^ Annual Meeting of the Association for Computational Linguistics, pp. 7839-7859, July 2020
2. Belinkov Y, Gehrmann S, Pavlick E. “Tutorial proposal: interpretability and analysis in neural NLP,” Proc of the 58^th^ Annual Meeting of the association for Computational Linguistics, pages 1-5, July 5, 2020.
3. Dunietz J. “The field of natural language processing is chasing the wrong goal,” MIT Technology Review, July 31, 2020.

**--------------------------------------**

**Question 21**: Also, the authors seem to suggest that HSCM can better handle out-of-vocabulary concept formulation and meaning understanding by way of semantic activation network. An illustration of this can again be provided via a comparison table or graphic visualization using a simple/concrete example that shows how HSCM is expected to handle an unseen term, "concept", or "semantic abstraction" versus skip-gram word2vec, for example. This is an opportunity to highlight how HSCM can provide superior human-like understanding compared to simple inference using word2vec.

## Response 21:

Thank you for this suggestion. We have included additional text to explain how various ambiguities (e.g., out-of-vocabulary words) can be handled by the design.

The agent-based architecture described in the design allows the system to incorporate diverse algorithms to map to nodes within the HSCM. For the case of out-of-vocabulary words, an agent would be responsible for extracting relevant features including embeddings, morphological features, edit distance to known words, and soundex signatures that can be integrated using a classifier. Thus, the agent architecture provide the mechanism for integrating multiple features using several analysis perspectives. We have emphasized in the text that the described architectural design does not exclude using deep learning or any other algorithms for the problem of symbol-to-symbol mappings defined within by HSCM.

**--------------------------------------**

**Question 22**: Perhaps HSCM is able to leverage embedding-based representations at its various levels, which gives users of the framework best of both worlds. This aspect could also be elaborated on.

## Response 22:

Thank you for this suggestion. In the Discussion, we re-iterate that the design is an overarching framework that provides semantic precision, expandability, knowledge integration, and transportability (which are limitations of current deep learning transformer methods). The implementation of the framework can use deep learning methods / features (e.g., embeddings) to facilitate mappings at any node at any level of abstraction within the HSCM. Embeddings can also be used to guide the activation of hypothesis nodes during the predictive coding process. This is suggested from recent neuro-cognitive studies showing a strong relationship between deep learning features (e.g., word-to-vec and internal nodes of BERT and GPT-3) and areas of activation in the cortical surface of the brain [Caucheteux 2020, Caucheteux 2021]. We have added these points in the Discussion section of the paper submission. Finally, as you have suggested, we have expanded this discussion to the general idea of hybrid neuro-symbolic architectures which aim to utilize the complementary strengths of deep learning and symbolic AI paradigms [Garnelo 2019].

Caucheteux C, Gamford A, and King J-R. “GPT-2’s activations predict the degree of comprehension in the human brain, bioRxiv doi: https://doi.org/10.1101/2021.04.20.440622, June 10, 2021.

Caucheteux C and King J_R. Language processing in brains and deep neural networks: computational convergence and its limits,” bioRxiv preprint doi: <https://doi.org/10.1101/2020.07.03.186288>, July 4, 2020.

Garnelo, M. and Shanahan, M. (2019) ‘Reconciling deep learning with symbolic artificial intelligence: representing objects and relations’, *Current Opinion in Behavioral Sciences*. Elsevier Ltd, 29, pp. 17–23. doi: 10.1016/j.cobeha.2018.12.010.

**--------------------------------------**

**Question 23**: Finally, I believe that the work should be presented for publication after some very basic observations and outcomes are gathered and evaluated from an initial prototype.

## Response 23:

Thank you for this suggestion.  We have tried to remain free of implementation details, as we feel it may distract the reader from the flow of presenting the broader concepts.  We did not want readers to fixate on implementation issues.  Rather, the paper’s main goal is to present the medical informatics community an overview of the principles and paradigms that should be considered if free-text understanding is to be ultimately achieved across a wide spectrum of clinical report types.  Since cognitive architectures for NLU are relatively unexplored within the field, this paper introduces the topic at the level of “What” and “Why”, but not “How”.  Thus, the proposed architecture introduces the concepts of semantic priming, semantic frames, compositional semantics, situational ontologies, discourse micro-theories, hierarchical predictive coding, and distributed agent-based processing. We feel it is out of the scope of this paper to provide an understandable level of detail related to implementation that is required by readers.  Note however, that the description of the parser includes a detailed step-by-step account of how the design analyzes a sentence during execution.  These steps follow exactly how our current prototype operates.   We will be submitting two follow-up papers that will describe in detail the implementation of the described design (see Future Directions Section).

**--------------------------------------**

**Question 24**: Overall, the paper outlines some powerful ideas that seem to have the potential to "truly" solve NLU for the medical domain, however it must be presented in the context of what current medical NLP/IR systems based on deep-learned representations do not yet have the capacity to solve, and by showing via example or anecdote from the HSCM framework, as to how a system based on this framework has a better chance of solving that specific NLU problem.

## Response 24:

We have added a paragraph in the Introduction section to highlight the current deficiencies of deep learning data driven methods. We include two recent references from the literature describing the current limitations of deep learning methods [Bender 2020] and the need to integrate knowledge into language understanding systems [Bisk 2020]. This argument is supported by well-established theories in which language understanding requires both appropriate interpretation of language signals (i.e., the text) and knowledge of world situations [Chomsky 1980, Bender 2015]. We also provide an example from our experience processing smoking behavior descriptions on the need for situational knowledge for interpreting author communicative intent.

Bender EM and Koller A. Climbing toward NLU: on meaning, form, and understanding in the age of data,” Proceedings of the 58th Annual Meeting of the Association for Computational Linguistics, (Best Theme Paper), Pages 5185-5198, July 2020.

Bisk Y, Holtzman A, Thomason J, Andreas J, Bengio Y, Chai J, Lapata M, Lazaridou A, May J, Nisnevich A, Pointo N, and Turian J. “Experience grounds language,” Proceedings of the 2020 Conference on Empirical Methods in Natural Language Processing, pp 8718-8735, November 16-20, 2020. Association for Computational Linguistics.

Chomsky N. Language and Learning: The Debate between Jean Piaget and Noam Chomsky. Harvard University Press, 1980.

# End of Questions ----------------------------------
